# Supplementary material for: COP27 Climate Change Conference: Urgent action needed for Africa and the world
Source: eClinicalMedicine. 2022 Oct 18;53:101721. doi: 10.1016/j.eclinm.2022.101721 (PMC9716324; doi:10.1016/j.eclinm.2022.101721)
Supplement: Appendix [file mmc1.pdf]

Title
Acta Paediatrica
Advanced Genetics
Advances in Nursing Science
Advances in Nutrition
African Health Sciences
African Journal for Physical Activity and Health Sciences
African Journal of Clinical and Experimental Microbiology
African Journal of Current Medical Research
African Journal of Gastroenterology and Hepatology
African Journal of Infectious Diseases
African Journal of Laboratory Medicine
African Journal of Primary Health Care & Family Medicine
African Journal of Reproductive Health
Afrimedic Journal
Afro-Egyptian Journal of Infectious and Endemic Diseases
Age and Ageing
Alcohol and Alcoholism
Allergy
Allergy: European Journal of Allergy and Clinical Immunology
Alpha Psychiatry
AlQalam Journal of Medical and Applied Sciences
American Journal of Clinical Pathology
American Journal of Epidemimology
American Society of Microbiology
Anatolian Journal of Cardiology
Anatomy Journal of Africa
Annales Africaines de Medecine
Annals of African Surgery
Annals of Global Health
Annals of Health Research
Annals of Oncology
Annals of the Rheumatic Diseases
Archives of Disease in Childhood
Archives of Neurology & Psychiatry (1919-1959)
Archives of the Turkish Society of Cardiology
Australian Journal of Rural Health
Babcock University Medical Journal
Bayero Journal of Medical Laboratory Science
Belgian Journal of Medicine and Health Care
BJOG: An International Journal of Obstetrics and Gynaecology
BMJ
BMJ Evidence-Based Medicine
BMJ Global Health
BMJ Health & Care Informatics
BMJ Innovations
BMJ Leader
BMJ Medicine
BMJ Military Health
BMJ Nutrition, Prevention & Health

Title
BMJ Open
BMJ Open Ophthalmology
BMJ Open Quality
BMJ Open Sport & Exercise Medicine
BMJ Paediatrics Open
BMJ Quality & Safety
BMJ Sexual & Reproductive Health
BMJ Supportive & Palliative Care
BMJ Surgery, Interventions, & Health Technologies
Borno Medical Journal
Bosnian Journal of Basic Medical Sciences
Brain
Brain Communications
British Dental Journal
British Journal of Clinical Pharmacology
British Journal of Sports Medicine
Canadian Journal of Respiratory Therapy
Cardiologia Croatica
Cardiovascular Diagnosis and Therapy
Cardiovascular Research
Caribbean Medical Journal
Central African Journal of Medicine
Cerrahpaşa Medical Journal
Clinical Medicine
Crohn's & Colitis 360
Curationis
Cureus Journal of Medical Science
Current Developments in Nutrition
Danish Medical Journal
Disability, CBR and Inclusive Development journal
Dutch Journal of Medicine (Nederlands Tijdschrift voor Geneeskunde)
East African Medical Journal
Eastern Mediterranean Health Journal
eBioMedicine
eClinicalMedicine
Emergency Medicine Journal
Environmental Epigenetics
EP Europace
Equine Veterinary Education
Equine Veterinary Journal
Ethiopian Journal of Health Sciences
Ethiopian Journal of Pediatrics and Child Health
Eurasian Journal of Medical Advances
Eurasian Journal of Medical Investigation
Eurasian Journal of Medicine and Oncology
European Heart Journal
European Heart Journal - Acute Cardiovascular Care
European Heart Journal - Cardiovascular Imaging

Title
European Heart Journal - Case Reports
European Heart Journal - Digital Health
European Heart Journal - Quality of Care and Clinical Outcomes
European Journal of Cardio-Thoracic Surgery
European Journal of Cardiovascular Nursing
European Journal of Hospital Pharmacy
European Journal of Preventive Cardiology
European Science Editing
Evidence-Based Mental Health
Evidence-Based Nursing
Evidence-Based Nursing Research
Family Medicine and Community Health
Gaceta Sanitaria (Spanish)
General Psychiatry
Ghana Medical Journal
Global Health Action
Health Policy and Planning
Health Promotion Journal of Australia
Health Science Reports
Highland Medical Research Journal
Human Reproduction
IJQHC Communications
Indian Journal of Medical Ethics
Inflammatory Bowel Diseases
Innovation in Aging
International Journal of Gynecology & Obstetrics
International Journal of Health Policy and Management
International Journal of Laboratory Hematology
International Journal of Nursing Studies
International Journal of Older People Nursing
International Journal of Paediatrics and Child Health
International Journal of Pharmacy Practice
International Journal of Women's Dermatology
International Nursing Review
Internatonal Journal of Low-Carbon Technologies
JAMA
JAMA Cardiology
JAMA Dermatology
JAMA Health Forum
JAMA Internal Medicine
JAMA Network Open
JAMA Neurology
JAMA Oncology
JAMA Ophthalmology
JAMA Pediatrics
JAMIA Open
Journal de la Faculté de Médecine d'Oran
Journal of Advanced Nursing

Title
Journal of Child Health Care
Journal of Climate Change and Health
Journal of Dermatology for Physician Assistants
Journal of Dietitians Association of Nigeria
Journal of Epidemiology & Community Health
Journal of Global Health Reports
Journal of Global Health
Journal of Medical Association of Thailand
Journal of Medical Ethics
Journal of Medical Genetics
Journal of Medical Imaging and Radiation Sciences
Journal of Natural Sciences
Journal of Paediatrics and Child Health
Journal of Phytomedicine and Therapeutics
Journal of Public Health
Journal of Radiography and Radiation Sciences
Journal of the American Medical Informatics Association
Journal of the Norwegian Medical Association
Journal of the Royal Society of Medicine (JRSM)
Journal of Travel Medicine
Journal of Urban Ecology
Journal of Xiangya Medicine
Journals of Gerontology Series A: Biomedical Sciences and Medical Sciences
Kanem Journal of Medical Sciences
Khyber Medical University Journal
Laboratory Medicine
Libyan Journal of Medicine
Malawi Medical Journal
Mali médical
Mansoura Nursing Journal
Maternal & Child Nutrition
Medical Humanities
Medical Journal of Australia
Medical Journal of Australia
Medical Mycology
Medwave
Microbes and Infectious Diseases
National Medical Journal of India
Natural Sciences
NeuroOncology Advances
Neuro-Oncology Practice
New England Journal of Medicine
Nicotine & Tobacco Research
Nigerian Hospital Practice
Nigerian Journal of Medicine
Nigerian Journal of Natural Products and Medicine
Nigerian Journal of Paediatrics
Nigerian Journal of Pharmaceutical Research

Title
Nigerian Medical Journal
Nursing Inquiry
Nutrition & Dietetics
Occupational and Environmental Medicine
Occupational Medicine
Orapuh Journal
Oxford Open Energy
Oxford Open Immunology
Paediatric and Perinatal Epidemiology
Pakistan Journal of Medical Sciences
Palliative Medicine
Pediatric Infectious Disease Society of the Philippines Journal
Pediatric Nursing
Pharmacology Research & Perspectives
Philippine Journal of Otolaryngology Head and Neck Surgery
Public Health Challenges
Revue Africaine de Chirurgie et Spécialités
Rheumatology
Rheumatology Advances in Practice
RMD Open
RPS Pharmacy and Pharmacology Reports
Schizophrenia Bulletin
Schizophrenia Bulletin Open
Sierra Leone Journal of Biomedical Research
Sokoto Journal of Veterinary Sciences
Sonography
Stroke and Vascular Neurology
The American Journal of Clinical Nutrition
The Gerontologist
The Journal of Nutrition
The Lancet
The Lancet Child & Adolescent Health
The Lancet Diabetes & Endocrinology
The Lancet Digital Health
The Lancet Gastroenterology & Hepatology
The Lancet Global Health
The Lancet Haematology
The Lancet Healthy Longevity
The Lancet HIV
The Lancet Infectious Diseases
The Lancet Microbe
The Lancet Neurology
The Lancet Oncology
The Lancet Planetary Health
The Lancet Psychiatry
The Lancet Public Health
The Lancet Regional Health – Americas
The Lancet Regional Health – Europe

Title
The Lancet Regional Health – Southeast Asia
The Lancet Regional Health – Western Pacific
The Lancet Respiratory Medicine
The Lancet Rheumatology
Thorax
Transfusion Medicine
Tropical Journal of Pharmaceutical Research
Tunisie Medicale
Turkish Archives of Pediatrics
Turkish Journal of Biochemistry
Turkish Journal of Endocrinology and Metabolism
Turkish Thoracic Journal
Veterinary Anaesthesia and Analgesia
Veterinary Record
VOICE
West African Journal of Medicine
Western Journal of Emergency Medicine
WHO Bulletin
